# 68/m mit Dyspnoe, peripheren Ödemen und Gewichtsverlust: Vorbereitung auf die Facharztprüfung: Fall 134

**DOI:** 10.1007/s00108-021-01217-4

**Published:** 2022-01-11

**Authors:** C. Kimmich

**Affiliations:** Universitätsklinik für Onkologie und Hämatologie, Klinikum Oldenburg, Rahel-Straus-Straße 10, 26133 Oldenburg, Deutschland

**Keywords:** Systemische Amyloidose, Chronische Herzinsuffizienz, Hydropische Dekompensation, „Smoldering myeloma“, Monoklonale Gammopathie

## Prüfungssimulation

### Fallschilderung

Ein 68-jähriger Mann (180 cm, 75 kg) wird aufgrund ausgeprägter Dyspnoe NYHA III (New-York-Heart-Association-Stadium III) und thorakalen Druckgefühls bei Belastung von einem niedergelassenen Kollegen in der internistischen Notaufnahme vorgestellt. Der Patient berichtet von schleichend zunehmender Atemnot beim Treppensteigen seit mehreren Wochen und einer allgemeinen Abnahme der körperlichen Belastbarkeit seit einem halben Jahr. Die Unterschenkelödeme zeigten sich nach Beginn einer Therapie mit Torasemid 5 mg allenfalls leicht rückläufig. Weiterhin werden Reizhusten und eine störende Nykturie (3-mal/Nacht) beklagt. Letztere zeigt seit mehreren Monaten trotz Einnahme von Tamsulosin 0,4 mg keine Besserung.

Zudem erfolgte vor Kurzem eine Ösophagogastroduodenoskopie (ÖGD) zur Abklärung von Magenschmerzen und Inappetenz bei ungewolltem Gewichtsverlust von 5 kg. Hierbei zeigte sich lediglich eine Gastritis Typ C, die temporär mit Pantoprazol 40 mg behandelt wurde. Das Gewicht konnte seitdem stabil gehalten werden. Mittels Computertomographie (CT) des Abdomens wurde bereits eine abdominelle Raumforderung ausgeschlossen. Die Nierenfunktion ist mit einem Kreatininwert von 1,42 mg/dl (errechnete glomeruläre Filtrationsrate 52 ml/min pro 1,73 m^2^) leicht eingeschränkt.

## Prüfungsfragen

- An welche Differenzialdiagnosen denken Sie primär und warum? Worauf achten Sie in der körperlichen Untersuchung? Ergänzen Sie ggf. zielführend die Anamnese.
- Welche nächsten diagnostischen Schritte leiten Sie ein?
- Was fällt Ihnen im echokardiographischen Bild (Abb. 2) auf?
- Nennen Sie mögliche differenzialdiagnostische Ursachen einer Myokardhypertrophie. Welche Diagnose erscheint in Anbetracht der vorliegenden Informationen am wahrscheinlichsten? Welchen Laborwert sollten Sie trotz dieser Verdachtsdiagnose erneut kontrollieren?
- Welche weiteren diagnostischen Schritte leiten Sie ein?
- Nennen Sie weitere Punkte, die auf eine systemische Amyloidose hinweisen können.
- Was veranlassen Sie bei nachgewiesener monoklonaler Gammopathie?
- Wie lautet die bisherige Diagnose? Wie können Sie die systemische Amyloidose sichern?
- Wie behandeln Sie eine systemische Amyloidose? Was ist vor Einleitung einer Therapie zwingend erforderlich?

### Antworten

#### An welche Differenzialdiagnosen denken Sie primär und warum? Worauf achten Sie in der körperlichen Untersuchung? Ergänzen Sie ggf. zielführend die Anamnese

- Lungenarterienembolie (Dyspnoe, Reizhusten, Unterschenkelödeme)
- Myokardinfarkt/instabile Angina pectoris (Dyspnoe, epigastrische Schmerzen)
- **Hypertensive, ischämische oder amyloidosebedingte Kardiomyopathie **(Dyspnoe, Reizhusten, Nykturie, bekannter Hypertonus)
- Malignom: Bronchialkarzinom, akute Leukämie (Dyspnoe, Reizhusten, ungewollter Gewichtsverlust)
- Pneumonie (Dyspnoe, Reizhusten)
- Glomerulonephritis mit nephrotischem Syndrom (Unterschenkelödeme, Nykturie, chronische Niereninsuffizienz)

##### Der Fall.

Wichtige Punkte der **körperlichen Untersuchung:** Temperaturmessung (36,7 °C), Vitalparameter (Blutdruck 100/60 mm Hg; Puls 88 Schläge/min, rhythmisch, regelmäßig; Atemfrequenz 14/min), Mundschleimhäute rosig und reizlos, **gestaute Halsvenen in aufrecht sitzender Position**, peripherer Lymphknotenstatus unauffällig, regelrechter kardialer Auskultationsbefund, **abgeschwächtes Atemgeräusch basal rechts mit fehlender Atemverschieblichkeit in der Perkussion der Lunge**, Leber in Medioklavikularlinie 1 cm unter Rippenbogen weich palpabel, Abdomenbefund ansonsten unauffällig. **Symmetrische Unterschenkel- und prominente Knöchelödeme**

Gezielte Anamnese

- **Symptomorientiert: **Inappetenz, kein Brust- oder Oberbauchschmerz. keine Dysurie. kein schäumender oder dunkler Urin
- **Vegetativ:** ungewollter Gewichtsverlust von 5 kg, kein Alkohol- oder Nikotinabusus. Kein Fieber oder Nachtschweiß
- **Voroperationen und weitere Erkrankungen**: **Z.** **n. Hüfttotalendoprothese rechts 2010; im Anschluss tiefe Beinvenenthrombose (TVT). Z.** **n. Cholezystektomie**, Z. n. Gastritis Typ C, chronische Niereninsuffizienz G3b (Stadieneinteilung nach Kidney Disease: Improving Global Outcomes [KDIGO] 2012)
- **Familien‑/Sozialanamnese** mütterlicherseits positiv für Diabetes mellitus Typ 2. Keine Malignome oder Herz-Kreislauf-Erkrankungen in der Familienanamnese bekannt. Verheiratet, kinderlos, Angestellter mit hauptsächlicher Bürotätigkeit
- **Medikamente:** aktuell lediglich Torasemid 5 mg 1‑0‑0 und Tamsulosin 0,4 mg 0‑0‑1
- **Allergien:** keine bekannt

#### Welche nächsten diagnostischen Schritte leiten Sie ein?

**Figure 1:**
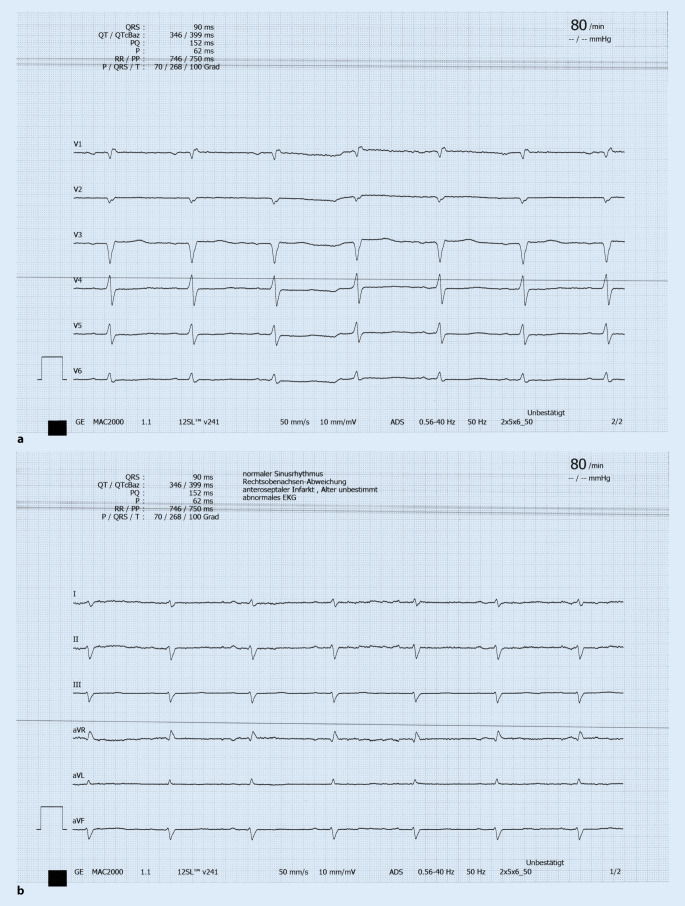

**Figure 2:**
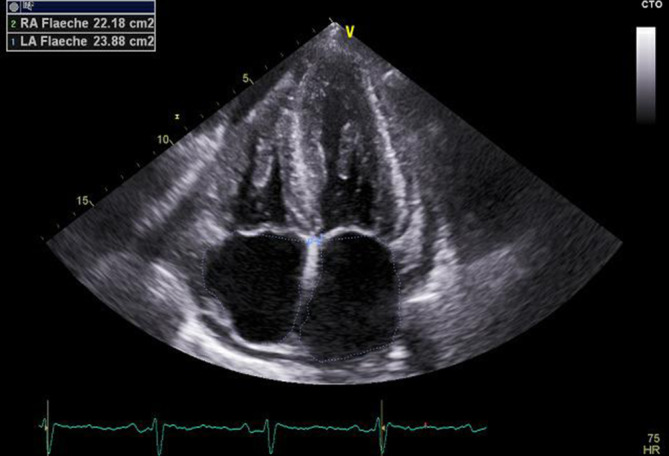

**Figure 3:**
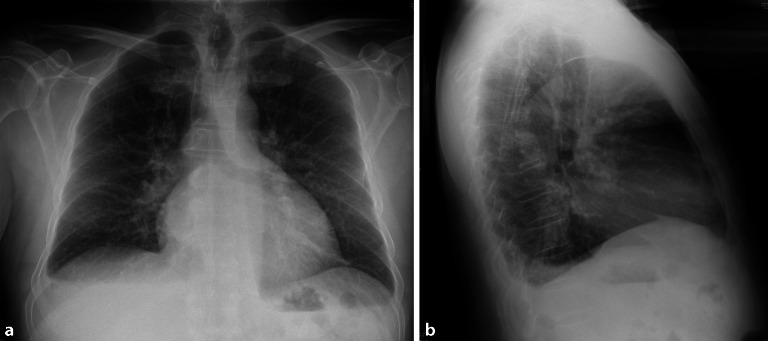

Labordiagnostik (Tab. 1), Elektrokardiogramm (EKG; Abb. 1a, b) und Echokardiographie (Abb. 2 und Video), Röntgenaufnahme des Thorax in 2 Ebenen (Abb. 3a, b). In Anbetracht der Anamnese mit Z. n. TVT und Dyspnoe wäre auch eine CT des Thorax mit Kontrastmittel möglich, wobei die chronische Niereninsuffizienz hierbei eine relative Kontraindikation darstellt.

**Table 1:** 

Chronische Niereninsuffizienz Grad 3b (Stadieneinteilung nach Kidney Disease: Improving Global Outcomes [KDIGO] 2012)
Verlängerung der International Normalized Ratio (INR)
Erhöhung der Cholestaseparameter: alkalische Phosphatase (AP), γ‑Glutamyltransferase (GGT)
Minimale Erhöhung der Alanin-Aminotransferase (ALT)
Latente Hypothyreose (thyreoideastimulierendes Hormon erhöht bei normwertigem freiem Thyroxin)
Erhöhtes hochsensitives Troponin mit 49 ng/l (Norm < 14 ng/l)
Hypokaliämie
NT-proBNP mit 6178 ng/l deutlich erhöht (altersspezifische Norm < 125 ng/l)
Urinstatus mit Ausnahme geringer Proteinurie unauffällig

*NT-proBNP* N-terminales pro-natriuretisches Peptid vom B‑Typ

- **Labordiagnostik** inklusive Differenzialblutbild, Troponin, Kreatinin, Harnstoff, Gerinnungsdiagnostik, Transaminasen und Cholestaseparameter, Bilirubin, ggf. D‑Dimere (in aktuellem Fall nicht erfolgt), Bestimmung des thyreoideastimulierenden Hormons und des N‑terminalen pro-natriuretischen Peptids vom B‑Typ (NT-proBNP), Urinstatus (Tab. 1)
- **Befund EKG** (Abb. 1a, b): Sinusrhythmus, 90 Schläge/min, Überdrehter Rechtstyp, R‑Verlust V1–V3, verzögerte R‑Progression über der Vorderwand mit R‑/S-Umschlag V5/V6, S‑Persistenz bis V6
- **Befund Röntgenaufnahme des Thorax in 2 Ebenen** (Abb. 3a, b): mittelständiges, nicht verbreitertes Mediastinum. Dilatierte Herzsilhouette mit angedeuteter Bocksbeutelform, passend zu einem Perikarderguss. Geringe Zeichen einer pulmonalvenösen Stauung. Keine umschriebenen Infiltrate. Minimale Pleuraergüsse beidseits, rechts mehr als links

#### Was fällt Ihnen im echokardiographischen Bild auf?

Septumbetonte Myokardhypertrophie, Vergrößerung beider Vorhöfe mit Perikardergusslamelle

Befund Echokardiographie:

- Linker Ventrikel normal groß, hochgradig hypertrophiert (Septumdicke 20 mm). Linksventrikuläre Kontraktilität normal bei einer Ejektionsfraktion von 60 %
- Strain-Analyse: longitudinale basale Hypokinese mit apikaler Aussparung („apical sparing“)
- Aorten- und Mitralklappe sowie Trikuspidalklappe fibrosiert mit Insuffizienz Grad I, keine Stenosen
- Systolischer Pulmonalarteriendruck 51 mm Hg + zentraler Venendruck. Restriktiv-reversible diastolische Funktionsstörung Grad III. Gute systolische Funktion des normal dimensionierten rechten Ventrikels
- Perikarderguss hämodynamisch nicht relevant, zirkulär, echoarm
- Maximale Ausdehnung 1,72 cm systolisch, enddiastolisch 1,42 cm vor rechtem Ventrikel

#### Nennen Sie mögliche differenzialdiagnostische Ursachen einer Myokardhypertrophie. Welche Diagnose erscheint in Anbetracht der vorliegenden Informationen am wahrscheinlichsten? Welchen Laborwert sollten Sie trotz dieser Verdachtsdiagnose erneut kontrollieren?

- **Speichererkrankung (kardiale Amyloidose**, Morbus Fabry, Hämochromatose etc.)
- Hypertensive Herzerkrankung (unwahrscheinlich, da Blutdruck normotensiv)
- Aortenklappenstenose (echokardiographisch ausgeschlossen)
- Hypertrophe Kardiomyopathie („apical sparing“ in Echokardiographie atypisch)
- Erneut zu kontrollierender Wert: **Troponin (stabil mit 45** **ng/l)**

##### Cave.

Trotz des hochgradigen V. a. eine Kardiomyopathie sollte ein erhöhter Troponinwert bei Vorstellung in einer internistischen Notaufnahme kontrolliert werden.

#### Welche weiteren diagnostischen Schritte leiten Sie ein?

**Figure 4:**
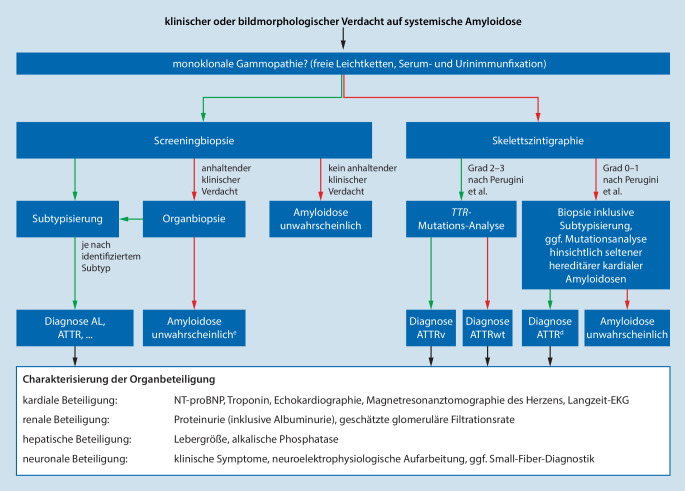

Diagnostischer Algorithmus in Abb. 4

Finden sich die in Tab. 1 angegebenen Befunde und besteht somit der V. a. eine systemische Amyloidose, sollte primär geprüft werden, ob eine **monoklonale Gammopathie** vorliegt. Hierzu gehören eine Messung der freien Leichtketten im Serum sowie eine Immunfixationselektrophorese von Serum und Urin.

Bei positivem Befund empfiehlt sich im nächsten Schritt die Durchführung einer Screeningbiopsie (subkutanes Fettgewebe, Speicheldrüse, Knochenmark oder oberer/unterer Gastrointestinaltrakt) zur Kongorotfärbung. Bei positivem Ergebnis ist im Anschluss zwingend eine Subtypisierung der Amyloidablagerungen erforderlich. Bei einem negativen Ergebnis und persistierendem V. a. Amyloidose sollte eine Zielorganbiopsie (Probenentnahme aus einem mutmaßlich betroffenen Organ) oder eine weitere Screeningbiopsie erfolgen.

Bei unauffälligem Screening auf das Vorliegen einer monoklonalen Gammopathie und V. a. eine kardiale Amyloidose empfiehlt sich die Durchführung einer Skelettszintigraphie mit ^99m^Tc-Diphosphono-Propandicarbonsäure (^99m^Tc-DPD), ^99m^Tc-Pyrophosphat (^99m^Tc-PYP) oder ^99m^Tc-Hydroymethylendiphosphat (^99m^Tc-HMDP). Bei Nachweis einer ausgeprägten kardialen Tracer-Anreicherung in der Spätphase (Perugini-Grad 2–3) ist die Diagnose der kardialen ATTR-Amyloidose (*ATTR* Amyloid aus Transthyretin) gesichert und eine Transthyretin(TTR)-Mutations-Analyse anzuraten. Bei negativer oder nur schwacher Tracer-Anreicherung [1] und fortbestehendem V. a. Amyloidose sollte eine Myokardbiopsie erfolgen.

##### Merke.

Wenn der V. a. eine systemische Amyloidose gestellt wird und eine histopathologische Diagnosesicherung indiziert ist, nutzen Sie eine Methode, die sowohl Sie als auch Ihr Pathologe regelmäßig anwenden. Alternativ kann die Vorstellung an einem Amyloidosezentrum erwogen werden.

##### Der Fall.

- **Kardiale Magnetresonanztomographie:** typisches Bild einer fortgeschrittenen Amyloidose mit restriktiver Kardiomyopathie, typischem „late gadolinium enhancement“ und vermehrtem extrazellulärem Volumen im T1-Mapping
- **Serologische Gammopathieabklärung** einschließlich Immunfixation von Serum und Urin, Messung der freien Serumleichtketten, Serumelektrophorese: Nachweis einer monoklonalen Gammopathie vom Immunglobulin-Gκ(IgGκ)-Typ mit IgG von 6,4 g/l und Erhöhung der freien κ‑Leichtketten im Serum auf 181 mg/l bei normwertigen freien λ‑Leichtketten von 15 mg/l
- Die **Urinuntersuchung auf Albuminverlust zur Abklärung einer Nierenbeteiligung bei systemischer Amyloidose oder Morbus Fabry** (entweder im 24 h-Sammelurin oder mittels Albumin/Kreatinin-Verhältnis) zeigt lediglich eine Mikroalbuminurie.
- Abdomensonographie (im aktuellen Fall nicht erfolgt, s. weiterer Verlauf)

##### Merke.

Bei konzentrischer linksventrikulärer Wandverdickung ohne hypertensive Herzerkrankung sollte an eine kardiale Amyloidose gedacht werden.

#### Nennen Sie weitere Punkte, die auf eine systemische Amyloidose hinweisen können

Nephrotisches Syndrom, Makroglossie, periorbitale Einblutungen, Karpaltunnelsyndrom, Spinalkanalstenose, Diarrhöen, sensomotorische und/oder autonome Neuropathie etc. (Tab. 2)

**Table 2:** 

Konzentrische linksventrikuläre Wandverdickung (≥ 12 mm) mit echoreicher Myokardtextur bei normo- oder hypotonen Blutdruckwerten
HFpEF, reduzierte longitudinale linksventrikuläre Funktion
Niedervoltage, atrioventrikulärer Block oder Vorhofflimmern im Elektrokardiogramm in Ruhe
Pleuraerguss (insbesondere isoliert rechtsseitig), Perikarderguss
Nephrotisches Syndrom (Albuminurie mit progredientem Nierenversagen)
„Harte“ Hepatomegalie mit Cholestaseparametererhöhung
Gewichtsverlust durch Inappetenz, rasches Sättigungsgefühl oder Diarrhöen und Malabsorption
Makroglossie, submandibuläre Weichteilvermehrung
Periorbitale Purpura und/oder oberflächliche spontane Hauteinblutungen
Atraumatische Bizepssehnenruptur, Karpaltunnelsyndrom oder Spinalkanalstenose (Prodromi für ATTR-Amyloidose)
Distal betonte aufsteigende sensomotorische Polyneuropathie mit Kribbelparästhesien und Taubheitsgefühl
Autonome Dysfunktion wie Herzfrequenzstarre, Gastroparese, orthostatische Hypotonie, erektile Dysfunktion
Glaskörpertrübungen

*ATTR* Amyloid aus Transthyretin, *HFpEF* „heart failure with preserved ejection fraction“ (Herzinsuffizienz mit erhaltener Ejektionsfraktion)

#### Was veranlassen Sie bei nachgewiesener monoklonaler Gammopathie?

- Knochenmarkdiagnostik inklusive zytologischer und histologischer Untersuchung, Durchflusszytometrie und Interphase-Fluoreszenz-in-situ-Hybridisierung (iFISH)

##### Der Fall.

CD138+-Befund: Nachweis einer monoklonalen Plasmazellpopulation mit κ‑Leichtketten-Restriktion und 15 %iger Knochenmarkinfiltration

- Ganzkörperschnittbildgebung zum Ausschluss eines symptomatischen multiplen Myeloms

##### Der Fall.

In diesem Fall Ganzkörper-CT ohne Nachweis von Osteolysen

##### Merke.

Ein Patient mit monoklonaler Gammopathie sollte eine Ganzkörperschnittbildgebung zum Ausschluss eines symptomatischen multiplen Myeloms erhalten.

#### Wie lautet die bisherige Diagnose? Wie können Sie die systemische Amyloidose sichern?

**Diagnose: „smoldering myeloma“ vom IgGκ-Typ mit hochgradigem V.** **a. systemische Leichtkettenamyloidose (AL-Amyloidose) mit kardialer Beteiligung**

- Sicherung mittels Screeningbiopsie (subkutane Fettgewebsaspiration/-biopsie, tiefe Rektumschleimhautbiopsie, Speicheldrüsenbiopsie, Knochenmark)
- Nachuntersuchung vorhandener Proben aus den letzten Monaten auf Amyloidablagerungen (z. B. Proben aus ÖGD oder Koloskopie)
- Zielorganbiopsie (insbesondere bei Unsicherheit in der Abgrenzung zu weiteren Kardiomyopathien)
- Bei V. a. eine systemische ATTR-Amyloidose könnte eine **Technetium-Szintigraphie (Skelettszintigraphie)** durchgeführt werden. Eine positive Tracer-Anreicherung in der Spätphase bei Abwesenheit einer monoklonalen Gammopathie ist beweisend für kardiale TTR-Amyloid-Ablagerungen.

##### Der Fall.

Es konnte im konkreten Fall Amyloid in der Rektumbiopsie sowie in der histologischen Knochenmarkuntersuchung nachgewiesen werden. Die subkutane Fettgewebsaspiration zeigte keine Amyloidablagerungen.

##### Merke.

Der Goldstandard zur Sicherung der Diagnose einer Amyloidose ist der histologische Nachweis mittels Kongorotfärbung.

##### Cave.

Auch wenn eine bevorzugte Screeningbiopsie (subkutane Fettgewebsaspiration) ohne Amyloidnachweis ist, sollten bei hochgradigem V. a. eine systemische Amyloidose weitere Screeningbiopsien oder eine Zielorganbiopsie durchgeführt werden.

#### Wie behandeln Sie eine systemische Amyloidose? Was ist vor Einleitung einer Therapie zwingend erforderlich?

Die Behandlung der systemischen Amyloidose folgt zwei Grundlagen:

- einerseits der Hemmung der Amyloidbildung mittels Reduktion oder Stabilisierung des zugrunde liegenden Vorläuferproteins,
- andererseits der supportiven Therapie bezogen auf die einzelnen Organmanifestationen.

Ursachentherapie:

- Dementsprechend ist eine **Subtypisierung der Amyloidablagerungen** zur **Bestätigung des Vorläuferproteins** zwingend erforderlich.
- Die Benennung der verschiedenen Amyloidosen erfolgt nach dem jeweiligen Vorläuferprotein [4].
- Bei Nachweis einer **systemischen ATTR-Amyloidose** sollte zudem eine genetische Testung auf eine hereditäre amyloidogene TTR-Genmutation erfolgen, da dies Einfluss auf verfügbare Therapien wie auch Relevanz für Familienangehörige von Betroffenen haben kann.
- Bei der Behandlung der **systemischen AL-Amyloidose** wird die zugrunde liegende Knochenmarkerkrankung mit antineoplastischen Therapien behandelt mit dem Ziel, die monoklonalen freien Leichtketten im Serum zu reduzieren bzw. zu beseitigen.
- Die Behandlung der **kardialen ATTR-Amyloidose** erfolgt mit dem TTR-Stabilisator Tafamidis, der das im Blut zirkulierende TTR-Tetramer stabilisiert und somit an der Ablagerung hindert.
- Für die Behandlung der **hereditären ATTR-Amyloidose** mit peripher-neuropathischer Beteiligung in frühen und intermediären Stadien stehen zudem seit 2018 zwei mit mRNA interferierende Medikamente (Patisiran und Inotersen) zur Verfügung.
- Die Behandlung der **systemischen AA-Amyloidose** zielt auf eine Reduktion des Entzündungseiweißes Serumamyloid A ab. In den meisten Fällen ist bei dieser Erkrankung eine entzündlich-rheumatische Erkrankung oder eine chronische Infektion/Entzündung ursächlich. Eine Behandlung erfolgt entsprechend entweder antiinflammatorisch oder durch eine Therapie der zugrunde liegenden Infektion/Entzündung.

##### Cave.

Keine Chemotherapie vor Bestätigung einer systemischen Leichtkettenamyloidose

Symptomatische Therapie:

- Die Evidenz zur supportiven Therapie der Organmanifestationen im Rahmen einer systemischen Amyloidose ist deutlich begrenzter.
- Bei renaler und/oder kardialer Beteiligung werden Salz- und Flüssigkeitsrestriktion empfohlen sowie Diuretika eingesetzt. Eine zu starke Vorlastsenkung sollte jedoch zur Vermeidung von arteriellen Hypotonien vermieden werden.
- Klassische Blutdrucksenker wie Angiotensin-converting-enzyme-Hemmer oder Angiotensin-II-Rezeptor-Subtyp-1-Antagonisten werden aus diesen Gründen insbesondere bei der systemischen AL-Amyloidose oftmals schlecht toleriert.

## Supplementary Information




